# Lectures on Diseases of the Nervous System

**Published:** 1885-05

**Authors:** 


**Lectures on Diseases of the Nervous System, Especially in Women.** By S. WEIR MITCHELL, M. D. Second edition. Revised and enlarged, with five plates. Philadelphia: Lea Brothers & Co. 1885.

This work from the pen of Dr. Weir Mitchell needs no words of commendation from us to the profession. In large and varied experience in this special class of diseases, and in accurate observation and analysis of nervous disorders, the author stands without an equal in this country, and whatever emanates from him claims the attention and confidence of the profession. This work is a valuable addition to the literature of the subject. Our American women furnish a fertile field of observation and study. The author has brought both ability and aptitude to his task, and presents a work which in its value is without a rival.

